# *Mycoplasma* and *Ureaplasma* carriage in pregnant women: the prevalence of transmission from mother to newborn

**DOI:** 10.1186/s12884-020-03147-9

**Published:** 2020-08-11

**Authors:** Avi Peretz, Oran Tameri, Maya Azrad, Shay Barak, Yuri Perlitz, Wadie Abu Dahoud, Moshe Ben-Ami, Amir Kushnir

**Affiliations:** 1 Clinical Microbiology Laboratory, The Baruch Padeh Medical Center Poriya, Hanna Senesh 818/2, Tiberias, Israel; 2 The Azrieli Faculty of Medicine, Bar Ilan University, Safed, Israel; 3 Department of Neonatology and Neonatal Intensive Care Unit, The Baruch Padeh Medical Center Poriya, Tiberias, Israel; 4 Department of Obstetrics and Gynecology, The Baruch Padeh Medical Center Poriya, Tiberias, Israel; 5 Research Institute, The Baruch Padeh Medical Center Poriya, Tiberias, Israel

## Abstract

**Background:**

*Mycoplasma* and *Ureaplasma* have been extensively studied for their possible impact on pregnancy, and their involvement in newborn diseases. This work examined *Mycoplasma* and *Ureaplasma* carriage among gravidas women and newborns in Israel, as well as associations between carriage and demographic characteristics, risk factors, pregnancy outcomes, and newborn morbidity rates.

**Methods:**

A total of 214 gravidas women were examined for vaginal pathogen carriage through standard culture and polymerase chain reaction assay. Pharyngeal swabs were collected from newborns of carrier mothers. Clinical and demographic data were collected and infected newborn mortality was monitored for 6 months.

**Results:**

Nineteen mothers were carriers, with highest prevalence among younger women. Pathogen carriage rates were 2.32% for *Mycoplasma genitalium* (*Mg*), 4.19% for *Ureaplasma parvum* (*Up*) and 2.32% for *Ureaplasma urealyticum* (*Uu*). Arab ethnicity was a statistically significant risk factor (*p* = 0.002). A higher prevalence was seen among women residing in cities as compared to villages. Thirteen (68%) newborns born to carrier mothers were carriers as well, with a higher prevalence among newborns of women delivering for the first time, compared to women that had delivered before. Infection rates among newborns were 20% for *Mg* (*p* = 0.238), 100% for *Up* (*p* < 0.01), and 28.5% for *Uu (p* = 0.058), with more male than female newborns being infected. No association was found between maternal carriage and newborn morbidity.

**Conclusions:**

Maternal *Mycoplasma* or *Ureaplasma* carriage may be associated with ethnicity and settlement type. Further studies will be needed to identify factors underlying these associations and their implications on delivery.

## Background

*Mycoplasma genitalium* (*Mg*), *Ureaplasma parvum* (*Up*) and *Ureaplasma urealyticum* (*Uu*) are members of the *Mycoplasmataceae* family, known as the smallest form of life in terms of size and genome length. The *Mycoplasma* genus is a part of a larger class called Mollicutes, which contains 200 species. These bacteria are common parasites in humans, mammals, reptiles, fish, arthropods and plants. In humans, these bacteria are part of the normal flora and are mainly present in the oropharynx, upper respiratory tract, and parts of a distal urinary tract and reproductive system. In the past, due to the presence of these bacteria in the normal flora of healthy populations, it was doubted whether they are pathogens. However, with time, it became firmly established that these bacteria play a role in sexually transmitted diseases (STDs) [1, 5].

Bacterial transmission from a mother to her child can occur via intrauterine infection, where the bacterium multiplies in the amniotic fluid and is then transmitted to the fetal lungs. This kind of infection could occur at the beginning of the pregnancy and when membranes are still intact. Alternatively, infection via the hematogenous route, involves the navel blood vessels from an infected placenta. A third means of transmission can occur via the respiratory and cutaneous membranes, while the baby passes through the birth canal [16]. *Mycoplasma* are being studied to examine their possible impact on the course and outcome of pregnancy (early labor contractions, early labor, early miscarriage, etc.) and their involvement in newborn diseases.

*Ureaplasma* spp. were only first identified around the beginning of the new millennium; therefore, it is safe to assume that studies published prior to this period did not distinguish between the *Up and Uu* species [4, 7]. In previous studies, the highest infection rate (40–80%) was found in the vagina and cervix of asymptomatic women. In addition, these bacteria were the most prevalent among all bacteria found in the amniotic fluid and placenta [16], Later studies that distinguished between the two species found a higher prevalence of *Up* than *Uu* among pregnant versus non-pregnant women [4, 7]. In regards to newborns, prospective cohort studies demonstrated that infection with *Uu* slows fetal growth and is associated with low birth weight, a risk which is high especially in cases of bacterial vaginosis [14, 15]. Many works have shown that *Ureaplasma* causes congenital and neonatal pneumonia, with some describing cases of neonatal bacteremia leading to severe pneumonia, pulmonary hypertension and mortality [16]. Most studies have not found any association between *Mg* and obstetric labor complications [1]. A case-control study conducted in Guinea-Bissau, involving with 1041 women, found a 6.2% prevalence of *Mg* in the cervix 7 days after labor; implications for labor complications, such as silent birth, miscarriage, preterm labor, and small fetal weight, were not found [6]. In a cohort study performed in London among 1216 women, early-stage pregnancy (< 10 weeks) urine samples and vaginal swabs examined by polymerase chain reaction (PCR) for presence of *Mg*, demonstrated minor pathogen presence (0.7%) and concluded that there is no reason to assume that it is a risk factor for pregnancy [11]. In another prospective study testing vaginal swabs obtained during week 21–25 of pregnancy, only four of the 124 women who had an early and spontaneous labor, were found to be *Mg* carriers [9].

The aim of this work was to investigate the carriage rate of these pathogens among laboring women in Israel and to characterize these women according to epidemiologic and demographic variables, to investigate the rate of infected newborns, and to identify risk factors. In addition, a possible correlation between carriage of pathogen and delivery outcomes and morbidity was assessed.

## Methods

### Patient characteristics

This study was performed at the Neonatal and Maternal Department at Poriya Medical Center, Israel, between June 2014 and January 2016. The department performs approximately 3500 deliveries each year. The study was approved by the institutional review board, approval no POR 31–2013. Consecutive admissions were offered study participation by the admitting nurse. Each participant signed an informed consent form before any study-related procedures were initiated. Clinical and demographic data were obtained from gravidas women and newborn medical records and subsequently, through contact with the community pediatrician. Data collected from mothers included age, residential environment (rural, urban), ethnicity, mode of delivery (vaginal birth, vacuum assisted or a C-section), and the total number of deliveries. Data collected from newborn charts included gender, delivery weight, and presence of complications up to 6 months after date of birth. Inclusion criteria for study entrance were gravida women aged 18–45, at any stage of labor, and any mode of delivery. For the newborns, inclusion criteria included newborns until the first week of their lives. Exclusion criteria included genetic disorders, birth defects, laboratory test-documented chorioamnionitis (culture origin from placenta, membranes, amniotic fluid) and antibiotic treatment 1 month before the study. Women with mental disabilities were not included in the study.

### Sample collection and molecular identification

The vaginal specimens were collected no later than 72 h after birth, into universal transport medium (UTM) (Copan, Brescia, Italy) by inserting 2–3 cm into the vagina and swabbing 360 degrees. In case of a positive result for single or multiple variants of the investigated pathogens, a sample was obtained from the newborn’s pharynx. The swabs were stored at room temperature until transfer to the lab, no later than 24 h after sampling, where they were tested for pathogen presence. DNA was extracted from the samples using the AccuPrep Genomic DNA extraction kit (Bioneer, Daejeon, Korea), according to the manufacturer’s instructions. Then, DNA was subjected to a multiplex PCR reaction using the Anyplex™ II kit) II STI-5 Seegene, Seoul, Korea).

### Statistical analysis

To examine the association between two unrelated categorical variables, the Fisher exact test was run for 2 × 2 tables and Fisher-Freeman-Halton test for tables larger than 2 × 2. To examine the association between two paired categorical variables, the McNemar’s test or exact McNemar’s test where run. t-test was used to examine the differences between continuous variables. Statistical significance was defined as *p*0.05 ≥. Statistical analyses were performed using SPSS 21.0.

## Results

### Prevalence rate of mycoplasma and Ureaplasma bacteria among gravida women

#### Carriage rates

**Fig. 1:**
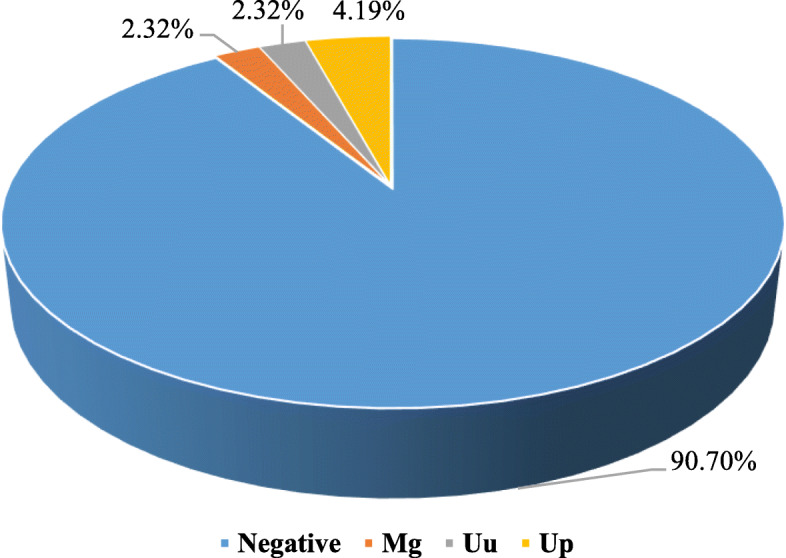
Percentages of positive specimens in the study, by pathogen and according to mother/newborns

A total of 214 women were sampled, among whom, 19 (9.3%) were found positive. Five (2.3%) participants were positive for *Mg,* 9 (4.2%) were positive for *Up,* and 5 (2.3%) were positive for *Uu* (Fig. 1).

#### Carriage rates in relation to age

Mean age of all women was 29.8 years. Mean age of the carrier women was 29.5 (22–38) years and mean age of the women that were found negative was 29.8 (19–45) years (*p* = 0.79). Out of 114 women between the ages of 18–29 years, 12 (10.5%) were found positive. Out of 93 women between the ages of 30–39 years, 7 ((7.5% were found positive. None of the women above the age of 40 years were carriers. No significant association was observed between age group and carriage rate (*p* = 0.51).

#### Carriage rates in relation to ethnicity

Sub-grouping into ethnicity groups identified 132 (61.8%) Jewish, 61 (28.5%) Arab, 6 (2.7%) Circassian, and 15 (7%) Druze women. Among Jewish women, 5 were found (3.8%) positive, while 11 Arab (18%), 2 Circassian (33.3%) and 1 Druze (6.7%) women were positive. The association between ethnicity and carriage rate was significant (*p* = 0.004). A post-hoc test revealed that carriage was more prevalent among Arab women (*p* = 0.02) and less prevalent among Jewish women (*p* = 0.006).

#### Carriage rates in relation to settlement type

A sub-grouping by residential setting demonstrated that 91 (42.7%) women lived in urban settings, and 122 (57.3%) lived in rural settings; no data were found for one woman. Among women living in urban settings, 10 (11%) were found positive. Among women living in rural settings, 8 (6.6%) were found positive, with no significant association between carriage rates and settlement type (*p* = 0.25) (Table 1).

**Table 1:** Summary of the demographic characteristics of mothers and PCR results

Characteristics	PCR assay results		***p***-value
	Positive (*N* = 19)	Negative (*N* = 195)	
Age (years) [n, (%)]			
**Mean**	29.5	29.8	0.791
**18–29** (*N* = 114)	12 (10.5%)	102 (89.5%)	0.510
**30–39** (*N* = 93)	7 (7.5%)	86 (92.5%)	
**≥ 40** (*N* = 7)	0	7 (100%)	
Ethnicity [n, (%)]			
**Jewish** (*N* = 132)	5 (3.8%)	127 (96.2%)	0.002
**Arab** (*N* = 61)	11 (18%)	50 (82%)	
**Circassian** (*N* = 6)	2 (33.3%)	4 (66.7%)	
**Druze** (*N* = 15)	1 (6.7%)	14 (93.3%)	
Living settings [n, (%)]			
**Urban** (*N* = 91)	10 (11%)	81 (89%)	0.250
**Rural** (*N* = 122)	8 (6.6%)	114 (93.4%)	

### Infection rate during labor and newborns’ characteristics

#### Total infection rates

**Fig. 2:**
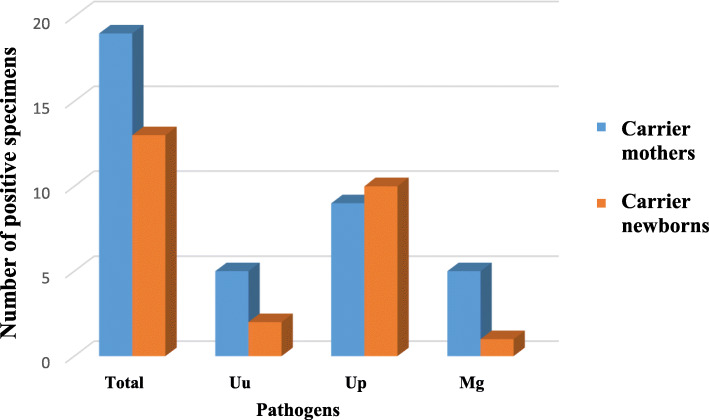
Number of infected maternal and newborn specimens

A total of 22 newborns were born to the 19 carrier women in labor; 3 (15.8%) were twin births. All the newborns were born alive and healthy. Among these newborns, 13 (59%) were found positive. One (20%) out 5 newborns born to women positive for *Mg*, was also positive for *Mg* with a non-significant change (*p* = 0.13), suggesting an association between maternal and newborn carriage of *Mg.* All 10 (100%) newborns (including a pair of twins) born to women positive for *Up* were also positive for *Up* (*p* = 1), and 2 (28.5%) of the 7 newborns born to women positive for *Uu* were also positive for *Uu* (*p* = 0.25) (Fig. 2).

#### Infection rates in relation to newborn gender

Fourteen newborns of carrier mothers were male; 9 (64.3%) were positive and 5 (35.7%) were negative. Eight females were born to carrier mothers; 4 (50%) were positive and 4 (50%) were negative. No significant association was observed between newborn gender and infection rates (*p* = 0.66).

#### Infection rates among twins

Out of the 3 pairs of twins that were born to women who were carriers, 2 pairs were born after in vitro fertilization (IVF) treatments. In one pair of twins, the mother and her male child were found to be positive for *Uu*, while the female child was negative for *Uu.* In another pair of twins, the mother and both her male and female twin babies were found positive for *Up.* In the third pair of male twins, the mother and one of her male children were found positive for *Uu.*

#### Infection rates in relation to gravidity

Out of the 19 carriers, 5 women were experiencing their first pregnancy, 4 (80%) whom had carrier newborns. Among the 14 multigravidas women, 8 (57.1%) had newborns who were positive. No significant association was observed between gravidity of carrier women and newborn infection rate (*p* = 0.60).

### Carriage implications for delivery outcomes and newborn health

#### Delivery outcomes

Out of the 19 carrier women, 16 (84.2%) had a regular delivery, 2 (10.5%) were vacuum-assisted, and one (5.3%) was an emergency Caesarean section. All the deliveries among women positive for *Mg* were regular. Among women positive for *Up*, 2 (22.2%) delivered with vacuum-assisted and 7 (77.8%) had a regular delivery. Among women positive for *Uu,* one (20%) delivered via a Caesarean section and 4 (80%) in regular deliveries. Fourteen (73.6%) deliveries were on the scheduled date and 5 (26.3%) were preterm - 1 (20%) of a woman positive for *Mg*, 1 (11.1%) of a woman positive for *Up,* and 3 (60%) of women positive for *Uu*. No significant association was observed between week of delivery of carrier women and newborn infection type (*p* = 0.18).

In the current work, it was found that among women positive for the bacteria, 25% of the deliveries were preterm (before week 37), 2 (10.5%) were performed with the assistance of a vacuum, and one (5.3%) delivery resulted in an urgent Caesarean section (Table 2). No significant association was observed between type of delivery of carrier women and newborn infection type (*p* = .433).

**Table 2:** Delivery outcomes in accordance with maternal type of bacterial carriage

Characteristic	***Ureaplasma*** and ***Mycoplasma*** carriers, n (%) (***N*** = 19)			***p-***value
	***U. urealyticum*** (*N* = 5)	***U. parvum*** (*N* = 9)	***M. genitalium*** (*N* = 5)	
Week of delivery [n, (%)]				
**Week + 37** (*N* = 14)	2 (40%)	8 (88.9%)	4 (80%)	0.179
**Preterm** (*N* = 5)	3^a^ (60%)	1 (11.1%)	1 (20%)	
Type of delivery [n, (%)]				
**Regular** (*N* = 16)	4 (80%)	7 (77.8%)	5 (100%)	0.433
**Vacuum** (*N* = 2)	0	2 (22.2%)	0	
**Emergency Caesarian** (*N* = 1)	1^b^ (20%)	0	0	

^a^Two preterm deliveries resulted in twin births - one was found positive for *Uu* and the second negative

^b^Twin delivery resulted in an emergency Caesarian section - one was found positive for *Uu* and the second negative

#### Newborn characteristics

Mean weight of the newborns born to carrier mothers was 3052 g; mean weight of newborns that were not infected was 3124.4 g and mean weight of all newborns who were positive for the presence of the pathogen was 3016.9 g. Tables 3 and 4 present weight in relation to maternal pathogen carriage and in relation to newborn pathogen carriage. Three (13.6%) newborns weighed under 2500 g at birth, 2 (66.7%) of whom were positive (one for *Uu* and one for *Up*). Neither of the carrier newborns was born under 1500 g. No significant association was observed between newborn weight and infection rate (*p* = 0.42) or in mothers (*p* = 0.87). During the six-month follow-up period, no morbidity was recorded by the attending pediatrician in children born to carrier mothers.

**Table 3:** Delivery weight versus maternal bacterial infection subspecies

Weight/Pathogen	***U. urealyticum*** (***N*** = 7)	***U. parvum*** (***N*** = 10)	***M. genitalium*** (***N*** = 5)
**Mean (g)**	3052.05 (total)		
	2845.7	3031.0	3264.0
**≤ 2500 g** (*N* = 3) [n, (%)]	1 (14.3%)	1 (10%)	1 (20%)
**> 2500 g** (*N* = 19) [n, (%)]	6 (85.7%)	9 (90%)	4 (80%)

*p* = 0.866

**Table 4:** Delivery weight versus carriage among newborns

**Characteristic**		***Positive*** **(*N*** **= 13)**			***Negative*** **(*N*** **= 9)**
		***U. urealyticum*** **(*N*** **= 2)**	***U. parvum*** **(*N*** **= 10)**	***M. genitalium*** **(*N*** **= 1)**	
**Weight** [n, (%)]	**Mean (g)**	3016.9 (total)			
		2465	3090.5	3190	3124.4
	**≤ 2500 g** (*n* = 3)	1 (50%)	1 (10%)	0	1 (11.1%)
	**> 2500 g** (*n* = 19)	1 (50%)	9 (90%)	1 (100%)	8 (88.9%)

*p* = 0.423

## Discussion

*Mg, Up* and *Uu* are part of normal human flora and found mainly in the urinary, reproductive, and respiratory tracts. The bacteria are sexually transmitted and are associated with STDs and other conditions, such as urethritis in men, pelvic inflammatory disease in women, and infertility. Although frequently present in amniotic fluid and placenta, these bacteria are not routinely examined in gravidas women, likely due to the complicated growth requirements and the need for PCR performance. In recent years, association between these pathogens and pregnancy has been investigated [1, 2, 5].

### Prevalence of carriage among pregnant women

Our current work, testing a population of 214 gravidas women in Galilee, Israel, showed a *Mg* carrier rate of 2.32%, *Up* carrier rate of 4.19% and *Uu* carrier rate of 2.32%. These results correspond with previous studies that found prevalence rates of 0.7–3.3% for *Mg* [13], and a higher prevalence of *Up* than *Uu* [4, 7].

### Risk factors for bacterial carriage

Bacterial carriage was slightly affected by maternal age, where age above 30 brought an increased risk of carriage, while no carriers were found among women above the age of 40. A possible reason could be a modification of the normal flora with age, due to physiological conditions or accumulation of antibiotic treatments during the course of life. Another feasible explanation is that partner multiplicity is more common in younger women, in accordance with other infections caused by other STD pathogens [12, 17].

Pathogen carriage frequency was statistically significantly higher (*p* = 0.002) in the Arab compared to the Jewish population. A higher carriage frequency was also found in Druze and Circassian populations as compared to the Jewish population; however, it was not statistically significant, may be due to a small sample size. No other works assessing infection rates in these ethnicities have been reported.

### Rates of mother-to-newborn bacterial transmission

Previous studies have shown that mothers-to-newborn transmission rates of *Ureaplasma* bacteria are between 18 and 88%; however as mentioned, distinction between species was made only in the beginning of the millennium [7]. The current work recorded an 28.5% transmission rate for *Uu* (*p* = 0.06), a 100% transmission rate for *Up* (*p* < 0.01) and a 20% transmission rate for *Mg* (*p* = 0.238). Estimation of the exact transmission rate will require further studies with larger cohorts.

### Risk factors for mother-to-newborn bacterial transmission

Very few reports examining factors that could potentially increase infection transmission from a mother to a newborn exist in the medical literature. While several works demonstrated differences in the carriage rates in different ethnicities, they didn’t focus on a specific group of newborns born to mothers who were carriers [1, 3, 10]. Although data extracted from the current study did not show statistical significance, they attest to a trend demonstrating that male newborns are more prone to infection than female newborns, more newborns of Jewish ethnicity are infected than newborns of Arab ethnicity, more newborns born to mothers living in urban settings are infected than newborns born to mothers living in rural settings, and more newborns born in a first delivery are infected as compared to newborns born to multiparous women.

### Bacterial carriage

Infection was not associated with an increase in morbidity in newborns in the 6 months after delivery. This finding lies in contrast with a previously published work that showed that *Ureaplasma* caused congenital and neonatal pneumonia, and some studies that described cases of neonatal bacteremia leading to severe pneumonia, pulmonary hypertension (PPHN), and mortality [3]. In addition, a meta-analysis performed in 2014, demonstrated a significant association between *Ureaplasma* presence in the lungs and development of bronchopulmonary dysplasia in preterm infants (< 36 weeks) and in newborns within 28 days of delivery, regardless of maternal age at birth [8].

## Conclusions

In conclusion, maternal pathogen carriage may be associated with ethnicity and settlement type. Further studies should be performed to identify the factors underlying these associations. Regarding the implications on delivery, *Ureaplasma* carriage seemed to correlate with higher percentages of preterm delivery and a vacuum-assisted delivery as compared to the average rates in Israel.

Further research should focus on identifying additional risk factors for bacterial carriage, such as fertility treatments, which has become increasingly common in recent years. In addition, investigations should assess the weight of socioeconomic factors and the differences between the different Galilee populations. Newborns should be monitored for extended periods of time in order to examine the possibility of an association between neonatal infection and long-term morbidity. Overall, the sample size should be enlarged for statistical power – particularly with regards to determination of the need for further screening of these pathogens in pregnant women.

## Data Availability

The datasets used and/or analyzed during the current study are available from the corresponding author at reasonable request.

## Abbreviations

Mg: *Mycoplasma genitalium*
Uu: *Ureaplasma urealyticum*
Up: *Ureaplasma parvum*
PCR: Polymerase Chain Reaction
